# Age-friendly AI: building bridges between technology and geriatric care

**DOI:** 10.1093/geroni/igaf093

**Published:** 2025-09-08

**Authors:** Thomas K M Cudjoe, Katherine Feemster, Sato Ashida, Peter Abadir, Phillip Phan, Nancy L Schoenborn

**Affiliations:** Division of Geriatric Medicine and Gerontology, Department of Medicine, Johns Hopkins University School of Medicine, Baltimore, MD, United States; The Green Fire Company, LLC, Bush, LA, United States; Department of Community and Behavioral Health, University of Iowa College of Public Health, Iowa City, IA, United States; Division of Geriatric Medicine and Gerontology, Department of Medicine, Johns Hopkins University School of Medicine, Baltimore, MD, United States; Department of Computer Science, Johns Hopkins Whiting School of Engineering, Baltimore, MD, United States; Carey Business School, Johns Hopkins University, MD, United States; Division of Geriatric Medicine and Gerontology, Johns Hopkins University, Baltimore, MD, United States; Division of Geriatric Medicine and Gerontology, Department of Medicine, Johns Hopkins University School of Medicine, Baltimore, MD, United States

**Keywords:** Artificial intelligence, Innovation, User-centered design, Stakeholder perspectives

Our recent conference, “Bridging Silos: Diverse Key Partner Perspectives on Leveraging AI to Improve the Care of Older Adults,” aims to advance the effort to plan for a coordinated future of data-driven decisions from a multidisciplinary perspective. In light of the rise in our aging populations, what has so far been found is that new technologies are most successfully adopted when they address not only a potential gap in service, but also the specific needs of the older adult patients and their caregivers, the physicians, nurses, and other health care staff who treat and support them.^1–3^ We believe that AI can integrate information across traditionally separate domains (including health records, pharmacy data, social services, home health, and many others) and help clinicians and caregivers address the 4Ms of the Age-Friendly Health System: what Matters, Medications, Mentation, and Mobility.^2^ The following conversations and presentations discussed the need to prioritize what matters most to older adults and their families, emphasizing independence and quality of life over strict adherence to clinical guidelines.

## Introduction

It is our position that there is a potential disconnect between what the older adult population needs from the health care system in the United States and what that system provides for them. We also fear that this situation risks further exacerbation for the future of geriatric medicine as modern technologies, such as Artificial Intelligence (AI) and large language models (LLMs), are integrated into that healthcare provision. This disconnect is further complicated by a fragmented delivery system and siloed specialty care sectors.

As both members of society and as patients, older adults face harsh and stressful transitions during the aging process. That is most apparent when they are faced with relinquishing their autonomy over their health and living conditions.^4^^,^^5^ They also do not exist as a monolith. They each have their own set of needs, as well as lifetimes of experience in an ever-changing world. Those experiences, their circumstances, families, educations, and racial/ethnic/cultural backgrounds are as varied as their biological ages. Meeting their needs and providing the care necessary for aging with dignity is one of the hallmarks of practicing geriatric medicine. The introduction of AI into this field brings the potential for improving health care for more of this population. It also brings with it several barriers to overcome to ensure its fair and equitable use.

As this population’s numbers grow over the coming years, so too will the need for fair and accurate data collection as the foundation for future innovations.^6–8^ There are historical health inequities and ageism biases that exist in the American health care system.^2^^,^^9^ This is an issue because many current health databases fail to include populations that experience health disparities or social and racial biases. Large Language Models are not only trained on this information, but some research models are also already building on them and even creating artificial data from them for future training and development needs.^9^^,^^10^

This technology’s effectiveness is dependent on the information provided for and by specific participant populations, geographic regions, cultures, and social determinants of health. To implement successful technological innovations, there will have to be conversations between the end users (the patients and providers) and the technical innovators (the developers and even the financial investors) during the design and development phases.^11–13^ Designing for and around the environments surrounding the technology will better serve to encourage any new system’s adoption—from the needs of both urban and rural clinics and hospitals, to the flexibility that is needed to fit into patients’ homes and lifestyles.

## The role of technology in closing gaps in care

The first presentation of this conference focused on the key considerations needed to be taken into account when developing health care technologies that are focused on older adults. During her talk, Dr Amanda Lazar focused on 3 areas of the NASSS (Non-adoption, Abandonment, Scale-up, Spread, and Sustainability) framework that are most relevant to older adults: the Value Proposition, the Condition, and the Adopter System.^3^

The *Value Proposition* addresses the question of the underlying purpose of technology from a business perspective. This should center on the value of the product to the end user (for example, the patient or care partner). It should also address the value and its achievement for the developer or the health system (the supplier) in undertaking or investing in the project.^3^

The *Condition* points to the need to understand the nature of the health care problem in focus, as well as any possible underlying comorbidities or psychosocial or cultural factors that may influence a successful adoption process. Specifically, older patients face several distinct challenges (described in further detail below) that need to be considered when designing technologies.^3^

The *Adopter System* reminds developers to be attentive to the intended end-user and their needs. End users may not only be the older patients but also their physicians, support staff, or other parts of the aging ecosystem of caregivers, programs, care centers, and their own families. Considerations of what the environmental needs (for in-home or clinical use) look like for a proposed new technology to be effective are also critical.^3^

User-centered design is an innovative approach that centers on incorporating many different aspects of technological design, human behavior, sociology, and even history.^14^^,^^15^ It takes effective communication skills from all those involved in the design process to effectively incorporate the needs and desires for the final products. Dr Lazar highlighted that the successful implementation of technologies (and their continued use) requires the active inclusion of, cooperation with, and participation of our older adult populations. The complexities faced by both designers and researchers in implementing modern technologies for older adults can also include certain, sometimes surprising considerations. Ageism is a particular concern, and is one that can be perpetuated by both physicians, when they begin to treat patients differently after certain diagnoses (ie, dementia), and by patients themselves, when they deny their own health risk or needs. Ageism is particularly relevant for AI model development since the data sources that many Large Language Models use for algorithm development and training are at risk of including this bias.

Consider the following examples of user-centered design projects provided by Dr Lazar through her own work, and how each could meet or miss the Value, Condition, and Adopter steps of the NASSS framework:

A recently published project involved implementing videoconferencing for patients with dementia-related diseases. During the development stage, she and her team focused on the accessibility of the software and mitigating what could be thought of as the more common technology issues of font size, removing unnecessary navigation buttons, and adding explanations for the ones that remained. During the project, though, they discovered that the greatest effort their older patients expended was not in managing the software program but in planning and preparing for the meetings and in their appearance and presentation during them.^14^^,^^16–18^ This highlights the importance of addressing context and environment for older adults, not simply the technology itself.

In 2020, The Health, Aging, and Technology Lab (THAT Lab) at the University of Maryland worked with a group of older adults in co-designing health-related projects (ie, medication alerts, exercising, monitoring vitals, and home maintenance).^8^^,^^10^^,^^14^^,^^16^^,^^17^^,^^19–21^ Participants went through a 5-step process: an initial one-on-one interview, a focus group where they discussed 6 of the most common “what-if” health and wellness scenarios identified during their initial interviews, a take-home journal assignment, a design workshop in a group setting, and a final follow-up one-on-one interview. Researchers found that the participants changed their approaches between the focus-group setting and the design workshop. During focus groups, the older adults were designing for others, generalizing for others they thought of as older and not themselves. The take-home journal assignment compelled them to think about their needs instead, leading to different end products.^17^^,^^18^^,^^22^ The perceived value and the actual need for interventions vary depending on the audience.

“Smart” pill bottles were designed and marketed for older adults. These bottles are meant to assist people with their medication schedules, providing alerts and timed dispensing. But a large-scale published review of over 50 000 patients showed that these “smart” bottles did not improve medication adherence. Participants shared their perspectives that the physical act of taking medication for a disease is uncomfortable (depending on the size and number of pills), and having visible and audible alarms and alerts served more to remind them of their disease or condition than they did the treatment. Dr Lazar opined during the presentation that the design of the bottles was also not discreet or conducive to being carried out in public for older adults who are active and have social lives.^23^^,^^24^ This highlighted the importance of developers needing to understand the full context of the barriers and the facilitators to adoption.

## Understanding the complexity of health care for older adults

Dr Cheryl Phillips gave the second presentation. During it, she broadly defined the issues surrounding the development and uptake of new technologies in health care. Dr Phillips explained the Age-Friendly Health System and the 4M framework of What Matters, Medication, Mentation, and Mobility. Along with being a holistic approach for geriatric care, the framework helps organize the thought process around the intersection of AI and aging and its multi-level complexity.

The design and integration of novel technologies is a multifaceted issue to address within several levels of the health care system. As health care providers, clinicians are beholden to the patients they serve and the health and financial systems in which they work. And the health systems, the insurance companies (ie, “the payors”), and private investment groups funding technological innovations are all entities that are important to the development of novel technologies, but each has its own objectives and benchmarks around safety, regulatory control, and profit.^25^^,^^26^ Challenges are found on many fronts:

- Payors often must bear the up-front costs for novel technology and may not be motivated to introduce changes to the already tried-and-tested status quo.
- Health systems are responsible for managing workflows and training employees, both aspects that are impacted by changing technology.
- The private investment groups and equity firms expect an efficient return on their investment and profits to fulfill their responsibilities to their shareholders and employees.

Perhaps unsurprisingly, the interplay of these relationships introduces additional tensions into patients’ journeys and compounds the difficulties for clinicians motivated to address the needs of their patients and communities. This added complexity makes it challenging to respond enthusiastically to accelerating technological growth and successfully implement new technologies.^2^^,^^27^ The health care community is still in the initial stages of adapting amid the surge in AI and is only just beginning to understand the ripple effects it could have in the field. At this point in time, there is still a window of opportunity to create standards of fairness and equity within the development process. Still, technological advancement can only succeed on a foundation of robust implementation where users want to use the products in their day-to-day lives.

Importantly, there is not just the need for trust between the patient and the clinician, but also between the patient and any new technology that is introduced into their care. AI holds many promising potential applications, but without patient buy-in and acceptance, its full potential cannot be reached.^11^ Older adults of all backgrounds, sociodemographics, and levels of experience have shared common concerns with technology taking up space and time in their medical care. Privacy considerations remain a significant source of that concern, particularly with the intrusive quality of some at-home medical devices and the loss of control over their private medical information to third parties.^28^^,^^29^ Additional difficulty arises when user interfaces are designed around parameters that do not consider the desires and requirements of the end user patients, resulting in real-world physical products being unwieldy or confusing, and thus not encouraging their continued use.

As both patients and members of society, older adults face a harsh transition into outsiders, seeing themselves become invisible as they relinquish their autonomy.^4^^,^^5^ Many have reported facing this transition and being met with more questions and concerns:

*“Has anyone asked me about my needs?”* Are medical practitioners talking to their patients, or are they instead talking about them in front of them to family members and caregivers?^3^^,^^6^^,^^9^

*“Can I afford this?”* This is not only a strictly financial question, though that is still a significant concern. Navigating health insurance plans, resources, and requirements is done over an ever-shifting landscape. At best, it is merely confusing, and at worst, it is life-altering. Is there any assistance available to organize and evaluate the choices?^26^^,^^27^^,^^30^^,^^31^

*“Do I have access to these services?”* The resources (training, personnel, and technology in particular) vary between rural and urban populations. Those gaps are only predicted to grow over the years. It is one reason for the push for AI and LLMs to be integrated into health care in the coming years.^4^^,^^9^

*“Is there trust here?”* There is not just the need for trust between the patient and the physician, but between the patient and any new technology introduced into their care. AI holds many promising potential applications, but without patient buy-in and acceptance, its full potential cannot be reached.^3^^,^^11^^,^^32^

To address these complexities, Age-Friendly Health Systems offers a framework to ground and unite the various stakeholder groups with intersecting interests and priorities. The 4Ms of What Matters, Medication, Mentation, and Mobility. There is an important reason “What Matters” is emphasized first—it is prominent as a reminder to keep the patient’s needs and perspective in mind.

## Interdisciplinary panel discussion

The conference included a discussion among 4 experts in their respective fields: Phillip Phan, PhD (Director, Networking & Mentoring Core, Johns Hopkins Artificial Intelligence & Technology Collaboratory for Aging Research [AITC], Carey Business School) represented the perspective of investors and payors, David Hurwitz, MD, MBA (Medical Director, Beacham Center for Geriatric Medicine) described a clinical perspective, Linda Burstyn (Director, Community Life Services, North Oaks Senior Living) represented the perspectives of older adults and care partners, and Casey Overby Taylor, PhD (Associate Professor, Medicine & Biomedical Engineering, Institute for Computational Medicine, Johns Hopkins School of Medicine) represented the perspective of technology developers. Nancy Schoenborn, MD, MHS (Associate Professor, Medicine, Division of Geriatric Medicine & Gerontology) moderated this section centered on the promises and perils of AI and the future of medicine.

Dr Hurwitz began by offering a practical example on the potential of AI from his own clinical experience as a geriatrician. In his practice, with just a single new patient, he has been known to spend several hours poring over both paper and digital medical records from multiple other clinicians to process years of a complicated medical history. Even more time is spent entering those findings into the electronic health record. He opined that this would be an ideal scenario for AI models to perform time-consuming administrative tasks. He also hoped that future developments would lead to more wearable technologies incorporating AI for data collation and transmission, offering further time savings and data interpretation.

Traditional health care models often prioritize disease management over older adults’ individual preferences and needs, leading to a mismatch between the care provided and the outcomes desired by patients and their families. Consider the implications in Dr Hurwitz’s example, where telemedicine combined with remote monitoring and AI-assisted decision support systems would improve data interpretation from multiple sources. For the clinicians, AI can simplify decision-making and allow the focus to remain on the patients. For the patients, treatment would mean not only medical interventions but an improved quality of life, particularly from the benefits of fewer stressful logistical considerations (ie, scheduling and travel to medical centers), improved communications, and potentially more options for care. Technology can be a method to make their lives happier, healthier, and more successful, in the form of better communication with their health care providers, in ways to better engage with their families, and in interacting with the world at large.

To reach those goals, panelists discussed the factors that must be considered during the adoption process. Foremost amongst those is the need to improve the monitoring and evaluation of the products, incorporating them not only during the design stage but also continuing through the planning and early product roll-out. This is particularly important when the products are implemented in populations not included in the training data sets. Additionally, new technology is typically not deployed efficiently at first, so having the capability built into the process to study the impacts and intermediate outcomes throughout the development process would ameliorate the potential disconnect between the product's engineering and its actual usefulness for its target audience.

That introduces the next factor that designers should consider during this process: having a clear definition of the unmet need. That definition needs to include the concept of the opportunity to solve the problem and who would benefit from the change. This process is described as the “3 walls of design”:

1. Evaluation and Validation: Is it a good product?
2. Effort vs Time: Would implementing the product require a significant change in workflows or dedicated training?
3. Value over Cost: What percentage of the population is this product going to help (is it a specialty product or intended for use in primary care)? Financially, can providers utilize Medicare coverage and break even during the implementation, or will they have to absorb the total cost up front?

And so, what will the future of technology and health care look like? Consider that there are already products on the market that exemplify the benefits of their incorporation. Devices such as the Alexa units function as personal assistants and can serve as outlets to the broader world for older adults. Ongoing interest in robotics has allowed for the development of new exoskeletons, which are a promising area for mobility improvements. Even autonomous cars or humanoid robots offer the opportunity for increased freedom and independence.

The future impacts of these technologies are promising, and these examples are only adjacent to the medical community; they were not necessarily developed with older adults as the target population. The final message of the panel section was one of hope, where work is done in collaboration, and everyone is motivated by ambitious visions for the future.

## Conclusion

A final point made during the panel discussion centered around the current state of medical training seems fitting to include here, though it veers from the modern necessities of technology and the potential impact of AI in clinical and research settings. Namely, it is that there seems to be a gap in current medical training as more new graduates from the younger generations are entering the workforce. Whether as a refresher or an introduction, there is a renewed need for teachers and programs to now make a point to implement the human touch and common-­sense approaches for their students—rethink their training to spend a little more time in the analog world instead of relying quite so heavily on technology alone. It is important, all agreed, to remember both how to teach and how to learn critical skills.

A central tenet in this discussion around how to bridge the silos of technology, research, and medicine is the importance of trust in health care. To achieve this foundational benchmark and simultaneously modernize health care to serve society better (and the older adult community in particular), developers and researchers must reframe what innovation means in medicine while at the same time including the scientific principles of measuring, monitoring, and evaluation.

Presenters and panelists agreed that knowing and understanding the end users of technology is paramount. When designing for patients, encouraging product adoption often means starting at the lowest level people are comfortable with and building up from that point. This method encourages participation in the process, and people are also more likely to adopt new processes if they can see the mitigating effects they have on the underlying issues they cope with in daily life.

As the field of geriatrics and gerontology continues to evolve, we must bridge the gaps between research, clinical practice, and technology to ensure that older adults receive the care they deserve. By facilitating real-time data sharing, decision support, and continuous feedback, AI technology can enable the realization of each of the “4Ms” in the Age-Friendly Health System. By working together across disciplines and sectors, we can create a more integrated, efficient, and compassionate health care system that truly meets the needs of our aging population.

## Data Availability

This article does not report data and therefore the preregistration and data availability requirements are not applicable.
